# Influence of Chymosin on Physicochemical and Hydrolysis Characteristics of Casein Micelles and Individual Caseins

**DOI:** 10.3390/nano11102594

**Published:** 2021-10-01

**Authors:** Chun-Chi Chen, Liang-Yu Chen, Wen-Tai Li, Ken-Lin Chang, Meng-I Kuo, Chao-Jung Chen, Jung-Feng Hsieh

**Affiliations:** 1 Department of Biological Science and Technology, School of Life Sciences, Longyan University, Longyan 364012, China; kennath1980@gmail.com; 2 Key Laboratory of Preventive Veterinary Medicine and Biotechnology, Longyan University, Longyan 364012, China; 3 Department of Food Science, Fu Jen Catholic University, New Taipei City 242, Taiwan; winniepig10@gmail.com (L.-Y.C.); 062998@mail.fju.edu.tw (M.-I.K.); 4 National Research Institute of Chinese Medicine, Ministry of Health and Welfare, Taipei City 112, Taiwan; lwt0220@nricm.edu.tw; 5 Institute of Environmental Engineering, National Sun Yat-Sen University, Kaohsiung City 804, Taiwan; klchang@mail.nsysu.edu.tw; 6 Ph.D. Program in Nutrition & Food Science, Fu Jen Catholic University, New Taipei City 242, Taiwan; jung9871@me.com

**Keywords:** chymosin, α_s_-casein, β-casein, hydrolysis, casein micelles

## Abstract

The effects of chymosin on the physicochemical and hydrolysis characteristics of casein micelles and individual caseins were investigated. Adding 0.03 units of chymosin/mL led to the casein micelles in skim milk coagulating after a 3 h incubation period at 30 °C. SDS–PAGE investigation showed that β-CN, κ-CN, α_s_-CN, and a portion of β-lactoglobulin (β-LG) in the milk supernatant fraction (MSF) were precipitated into the milk pellet fraction (MPF). The mean particle size of the MSF with chymosin decreased from 254.4 nm to 179.2 nm after a 3 h incubation period. Mass spectrometry and SDS–PAGE analysis suggested that chymosin hydrolyzed individual β-CN, κ-CN, and α_s_-CN, but not β-LG. Chymosin hydrolysis led to a decrease in the molecular weights of the hydrolyzed β-CN, κ-CN, and α_s_-CN. Particle size analysis indicated that there was no difference in the particle size distribution of hydrolyzed β-CN and α_s_-CN. Moreover, our outcomes demonstrated that the hydrolysis of κ-CN by chymosin occurs before that of β-CN and α_s_-CN.

## 1. Introduction

Milk is a complex mammalian liquid secretion of lipids, carbohydrates, lactose, and trace elements. The average composition of bovine milk is 4.5% lactose, 4.8% fat, 2.8% proteins, 87.2% water, and 0.7% ash [1]. The protein in milk consists of two protein collections: whey proteins (20%) and caseins (80%). The casein family, which is an intricate combination of several normal caseins, contains β-CN, κ-CN, and α_s_-CN at an individual ratio of 4:1:5 [2,3]. The molecular weights of β-CN, κ-CN, α_s1_-casein, and α_s2_-casein were 24.0, 19.0, 23.6, and 25.2 kDa, respectively [4]. The other type of milk protein, whey protein, consists of bovine serum albumin, β-LG, α-lactalbumin, and other constituents [5,6,7]. It is also a heterogeneous and polymorphic group of proteins, composed of 10% bovine serum albumin, 20% α-lactalbumin, 50% β-LG, 10% immunoglobulins, and <10% proteose peptones [8]. The molecular weights of β-LG, bovine serum albumin, and α-lactalbumin were 18.3, 66.3, and 14.2 kDa, respectively [9].

Milk is not only an important source of nutrition, but it is also important for industrial and commercial reasons. For example, milk is used in dairy-based products and other processed foods, such as cheese [10]. Cheese is a nutritious food and a significant wellspring of proteins, fatty acids, vitamins, and minerals [11]. Milk coagulation is an important stage in cheddar making, and its success is a significant boundary for obtaining high-quality cheese [12]. Various coagulants are utilized in cheese-making, but the most commonly used coagulant is chymosin [13]. In the process of making cheese, chymosin coagulates milk proteins enzymatically, and casein micelles are enzymatically destabilized during this process. Milk coagulation is a cycle in which liquid milk is turned into a viscoelastic semi-strong coagulum by an explicit chemical called chymosin [14,15].

Traditionally, chymosin (EC 3.4.23.4) is the main enzyme responsible for milk protein coagulation. Chymosin is a protease found in rennet that can precipitate milk proteins and promote the formation of curds during cheese making [16]. The enzyme works on milk κ-CN, somewhat separating these particles. This process causes an adjustment of micelle surface charge, increases their hydrophobicity, and encourages their conglomeration [17]. Furthermore, the natural substrate of chymosin is separated at the peptide connection between amino corrosive deposits as phenylalanine (105) and methionine (106). The chymosin induces the hydrolysis of κ-CN, which causes destabilization and aggregation of casein micelles [18]. In the process of making cheese from milk, when the cheese is aged, the hydrolysis of caseins prompts changes. Proteolysis is frequently considered to be the main biochemical pathway affecting the surface and flavor development [19].

In their 2012 study, Hsieh and Pan (2012) discussed the utilization of proteomic innovation to research the impacts of milk protein coagulation with chymosin [20]. The sample confirmed that the coagulation change of κ-CN is faster than the coagulation change of different caseins, such as β-CN, α_s_-CN, and a portion of whey protein. However, information about the relative susceptibility and coagulation of β-CN and α_s_-CN with chymosin treatment, apart from κ-CN, is lacking. Therefore, the purpose of this study is to investigate the effect of chymosin on the physicochemical and hydrolysis characteristics of casein micelles and individual β-CN, κ-CN, α_s_-CN, and β-LG.

## 2. Materials and Methods

### 2.1. Preparation of Skim Milk, Individual Caseins, and Chymosin

Raw milk was collected from healthy Holstein cows on a dairy farm in Taipei. Skim milk (29.9 mg/mL) was prepared by separating fat from raw milk by centrifugation (5000× *g*, 20 min), collected, and stored at 4 °C. Individual milk proteins, including β-CN (C6905), κ-CN (C0406), α_s_-CN (C6780), β-LG (L3908), and chymosin (R4877, 20 units/mg), were purchased from Sigma Chemical Co. (St. Louis, MO, USA). Chymosin was dissolved in a phosphate buffer (pH 6.8, 0.02 mM) prior to use. One unit of coagulation activity was defined as the amount of chymosin that coagulated 10 mL of milk per min at 30 °C.

### 2.2. Preparation of Skim Milk and Individual Casein Samples with/without Chymosin

Skim milk samples with/without chymosin (0.03 units/mL) were incubated at 30 °C for 0, 1, 2, and 3 h. Samples were then fractionated into the milk pellet fraction (MPF) and the milk supernatant fraction (MSF) by centrifugation at 5000× *g* for 20 min after incubation. One milliliter of each MSF sample was collected, and each MPF sample was redissolved in an equal volume (1 mL) of lysis solution containing 7 M urea, 2 M thiourea, and 4% 3-[(3-cholamidopropyl)dimethylammonio]-1-propanesulfonate prior to use. To examine the effects of chymosin on the hydrolysis of individual β-CN, κ-CN, α_s_-CN, and β-LG, 2 mg of each individual casein was dissolved in 0.02 mM phosphate buffer (pH 6.8). Each casein sample with 0.03 units of chymosin/mL was incubated at 30 °C for 0, 1, 2, and 3 h.

### 2.3. SDS-PAGE Analysis

After incubation, skim milk and individual caseins (including β-CN, κ-CN, α_s_-CN, and β-LG) with/without chymosin were analyzed by SDS-PAGE. In this study, samples were shown by using stacking gel (5%) and separating gel (12.5%). Then, 0.1 mL of each sample was mixed with 0.3 mL sample buffer (pH 6.8, containing SDS (2%), glycerol (10%), β-mercaptoethanol (5%), Tris-HCl (70 mM), and bromophenol blue (0.02%)). Each sample was heated at 95 °C for 5 min. The gel electrophoresis was run at 50 V for 30 min and then at 120 V for 2–3 h at constant current. After electrophoresis, Coomassie Brilliant Blue R-250 solution was used to stain the gels, which were imaged by an EPSON perfection V39 image scanner and then analyzed by Gel-Pro Analyzer software (version 4.0, Media Cybernetics, Inc., Rockville, MD, USA). SDS-PAGE profiles were used to evaluate the effect of chymosin on the hydrolysis of casein micelles and individual caseins.

### 2.4. Mass Spectrometry Analysis

To examine the effects of chymosin on the hydrolysis characteristics of individual caseins, individual β-CN, α_s_-CN, and β-LG samples with 0.03 units of chymosin/mL were hatched at 30 °C for 0 and 3 h. Matrix-assisted laser desorption/ionization time-of-flight mass spectrometry (MALDI-TOF-MS) was used to determine the molecular weights of the individual β-CN, α_s_-CN, and β-LG samples, which were analyzed on a Bruker Autoflex Speed MALDI-TOF-MS (Bruker Daltonics, Billerica, MA, USA) by measuring the mass-to-charge proportion (m/z) of the ionized analyte and recording the number of ions at each m/z value. The spectrum was acquired in linear positive mode at a laser frequency of 200 Hz. A method optimized for the mass range was established using the following parameters: acquisition range: 5000 to 35,000 Da.

### 2.5. Particle Size Analysis

To investigate the effects of chymosin on the physicochemical characteristics of casein micelles and individual caseins, skim milk, β-CN, and α_s_-CN with 0.03 units of chymosin/mL were hatched at 30 °C for 0 and 3 h. The particle size analysis was measured using a nanoparticle analyzer (SZ-100Z, HORIBA Instruments, Inc.). The particle size distribution spectra for the nanoparticles were recorded as diameter (nm) versus frequency (%), using the dynamic light scattering technique at a scattering angle of 90° and a temperature of 25 °C. The experiments on the sample were performed in triplicate.

### 2.6. Statistical Analysis

The results were analyzed using SAS^®^ version 9.4 (SAS Institute, Cary, NC, USA), and the data are displayed as the mean ± standard deviation. One-way analysis of variance was used to calculate significant differences between treatments. Each treatment was measured 3 times, and the statistical significance level was set to *p* < 0.05.

## 3. Results and Discussion

### 3.1. SDS-PAGE Analysis of Chymosin’s Effect on the Coagulation of Milk Proteins

**Figure 1:**
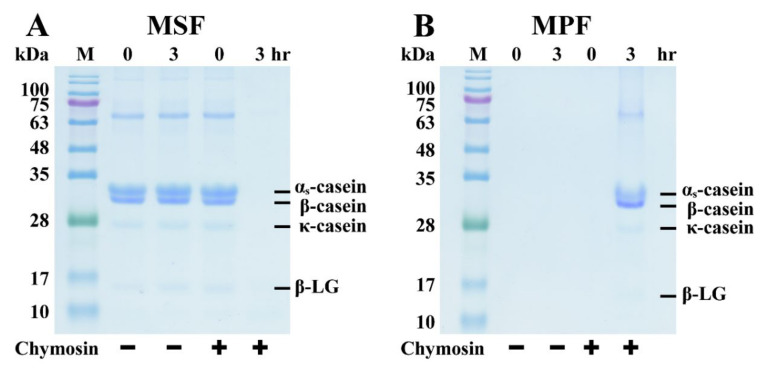
SDS-PAGE analysis of skim milk incubated with/without chymosin (0.03 units/mL) at 30 °C for 0 and 3 h. (**A**) Milk supernatant fraction (MSF); (**B**) milk pellet fraction (MPF). M = protein marker.

Skim milk samples with/without chymosin (0.03 units/mL) were incubated at 30 °C for 3 h, and the resulting MPF and MSF samples were analyzed by SDS-PAGE (Figure 1). The analysis results revealed that β-CN, κ-CN, α_s_-CN, and β-LG were the major milk proteins in skim milk. As shown in Figure 1A, the protein bands of β-CN, κ-CN, α_s_-CN, and a part of β-LG in chymosin-containing MSF disappeared after incubation for 3 h. However, these milk proteins were observed in the chymosin-containing MPF (Figure 1B). Furthermore, there were no significant changes in the protein bands of β-CN, κ-CN, α_s_-CN, and β-LG in the MSF and MPF without chymosin after the 3 h incubation period. These results indicate that some whey proteins and casein micelles were coagulated by chymosin into the MPF from the MSF. Horne (2006) reported that β-CN, κ-CN, and α_s_-CN in milk are consolidated into large colloidal structures called casein micelles [21]. These β-CN and α_s_-CN are connected with colloidal calcium phosphate, and κ-CN are assembled at the surface. Zhao and Corredig (2020) also reported that chymosin can precipitate casein micelles and promote the formation of curds during the cheese-making process [16]. Chymosin can cleave to κ-CN, resulting in a decrease in steric repulsion and net negative charge. It rapidly and specifically hydrolyzes κ-CN’s Phe105-Met106 bond, resulting in instability of casein micelles [22,23]. Hsieh and Pan (2012) suggested that these unstable casein micelles (β-CN, κ-CN, and α_s_-CN) and a part of whey protein such as β-LG are thought to be trapped in cheese curds [20]. Therefore, the above results demonstrate that chymosin induces the hydrolysis of κ-CN, which causes aggregation and destabilization of casein micelles. This result corresponded with that of Corredig and Salvatore [24].

### 3.2. SDS-PAGE Analysis of Chymosin’s Effect on the Hydrolysis of Individual Milk Proteins

**Figure 2:**
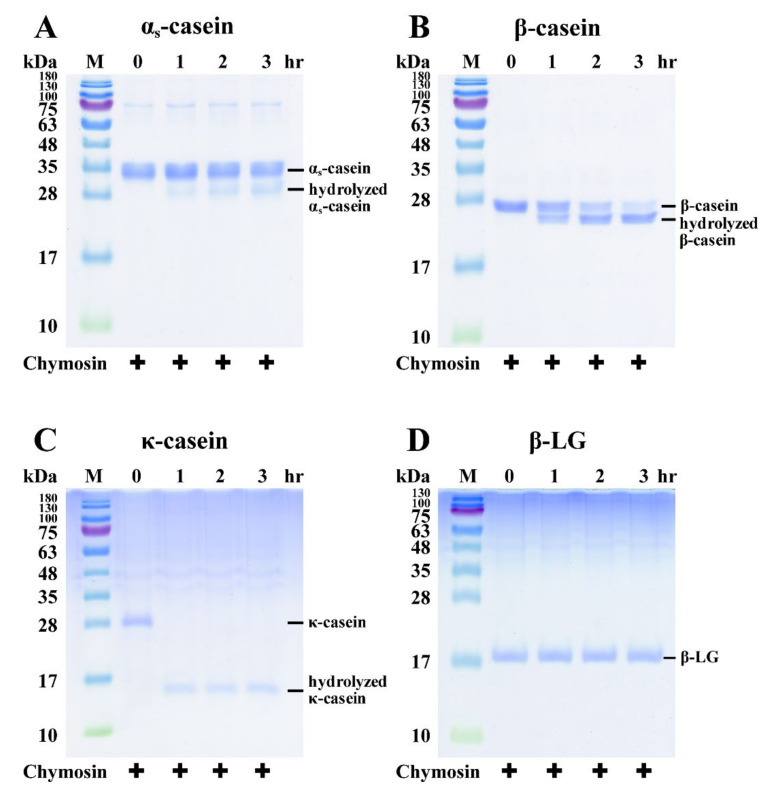
SDS-PAGE analysis of α_s_-CN, β-CN, κ-CN, and β-LG treated with chymosin (0.03 units/mL) at 30 °C for 0, 1, 2, and 3 h. (**A**) α_s_-CN; (**B**) β-CN; (**C**) κ-CN; (**D**) β-LG. M = protein marker.

**Figure 3:**
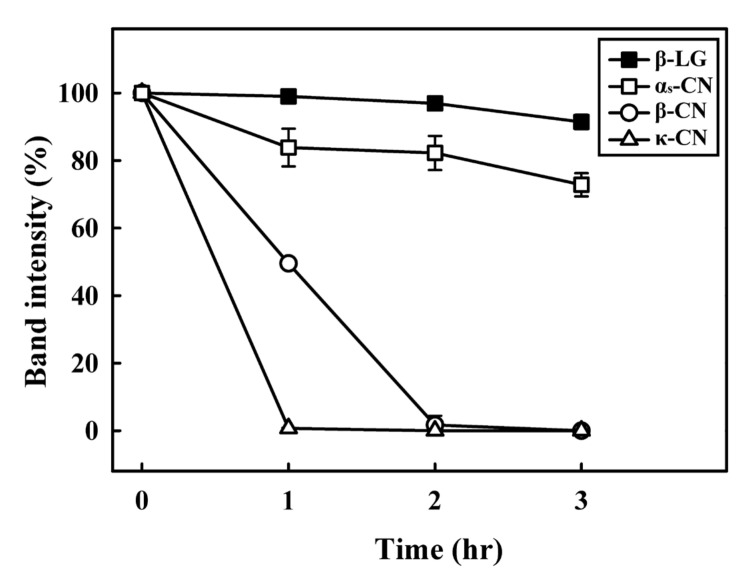
Densitogram corresponding to SDS-PAGE analysis of α_s_-CN, β-CN, κ-CN, and β-LG treated with chymosin (0.03 units/mL) at 30 °C for 0, 1, 2, and 3 h.

Individual β-CN, κ-CN, α_s_-CN, and β-LG samples with 0.03 units of chymosin/mL were incubated at 30 °C for 0, 1, 2, and 3 h, and the resulting samples were also analyzed by SDS-PAGE (Figure 2). As shown in Figure 2A, some α_s_-CN disappeared after a 1 h incubation period, while some hydrolyzed α_s_-CN was found by SDS-PAGE. The total intensities of α_s_-CN after 1, 2, and 3 h of incubation with chymosin decreased to 83.86 ± 5.59, 82.25 ± 5.06, and 72.84 ± 3.44%, respectively (Figure 3). These results indicated that chymosin hydrolyzed some α_s_-CN after 1 h incubation. Similar results were observed on chymosin-containing β-CN and κ-CN samples, and hydrolyzed β-CN (Figure 2B) and κ-CN (Figure 2C) were also found by SDS-PAGE after a 1 h incubation period. The total intensities of β-CN after 1, 2, and 3 h of incubation with chymosin significantly decreased to 49.54 ± 0.05, 1.72 ± 2.72, and 0.0 ± 0.0%, respectively (*p* < 0.05). The total intensities of κ-CN after 1, 2, and 3 h of incubation with chymosin decreased to 0.69 ± 0.17, 0.0 ± 0.0, and 0.0 ± 0.0%, respectively (Figure 3). Cooper et al. (2010) reported that chymosin is responsible for the specific cleavage of κ-CN [25]. Furthermore, chymosin also easily hydrolyzes peptide bonds in β-CN and α_s_-CN [26]. Therefore, these results also demonstrated that β-CN, κ-CN, and α_s_-CN were substrates for chymosin. However, we noticed no significant changes in the chymosin-containing β-LG sample after a 3 h incubation period (Figure 2D). The total intensities of β-LG after 1, 2, and 3 h of incubation with chymosin decreased to 99.02 ± 0.48, 96.93 ± 1.18, and 91.45 ± 2.09%, respectively (Figure 3). Therefore, β-LG is considered to be a poor substrate for chymosin.

### 3.3. MALDI-TOF-MS Analysis of Chymosin’s Effect on the Hydrolysis of Individual Milk Proteins

**Figure 4:**
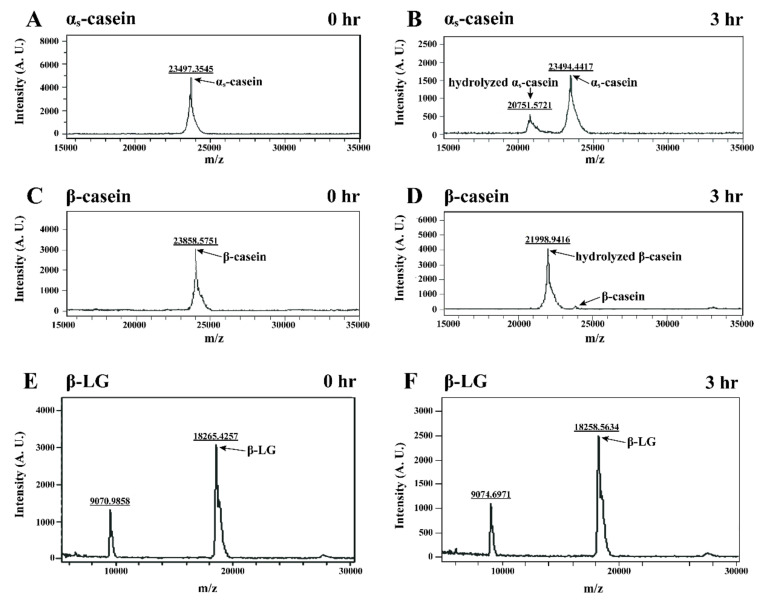
Mass spectrometry analysis of α_s_-CN, β-CN, and β-LG treated with chymosin (0.03 units/mL) at 30 °C for 0 and 3 h. (**A**) α_s_-CN for 0 h; (**B**) α_s_-CN for 3 h; (**C**) β-CN for 0 h; (**D**) β-CN for 3 h; (**E**) β-LG for 0 h; (**F**) β-LG for 3 h.

κ-CN is known to be a good substrate for chymosin, and κ-CN is hydrolyzed by chymosin to frame two peptide-like response items, which were the result of the specific cleavage of the Phe105-Met106 bond of κ-CN [27]. According to the results above, chymosin also hydrolyzed β-CN and α_s_-CN. Horneffer et al. (2007) indicated that MALDI-TOF-MS can be used to determine the molecular weights of proteins and is suitable for food research [28]. Therefore, individual β-CN, α_s_-CN, and β-LG samples with 0.03 units of chymosin/mL were incubated at 30 °C for 0 and 3 h, and the molecular weights of the resulting samples were analyzed by MALDI-TOF-MS. As shown in Figure 4A,C,E, the molecular weights of β-CN, α_s_-CN, and β-LG were 23858.5751 Da, 23497.3545 Da, and 18265.4257 Da, respectively. Haginaka (2000) reported that the molecular weights of β-CN, α_s_-CN, and β-LG were 24.0 kDa, 23.6 kDa, and 18.3 kDa, respectively [29]. However, the molecular weights of hydrolyzed β-CN and α_s_-CN induced by chymosin after 3 h incubation were decreased to 21998.9416 Da and 20751.5721 Da, respectively (Figure 4B,D). Kim et al. (2004) reported that chymosin hydrolyzed the Leu192-Tyr193 and Phe23-Phe24 bonds in β-CN and α_s_-CN, respectively [26]. These processes resulted in a decrease in the molecular weights of the hydrolyzed β-CN and α_s_-CN. However, there were no significant changes in the molecular weights of β-LG (18258.5634 Da) after a 3 h incubation with chymosin (Figure 4F). This result demonstrated that β-LG was not hydrolyzed by chymosin after 3 h of incubation.

### 3.4. Particle Size Analysis of Milk Proteins and Individual Milk Proteins Treated with Chymosin

**Figure 5:**
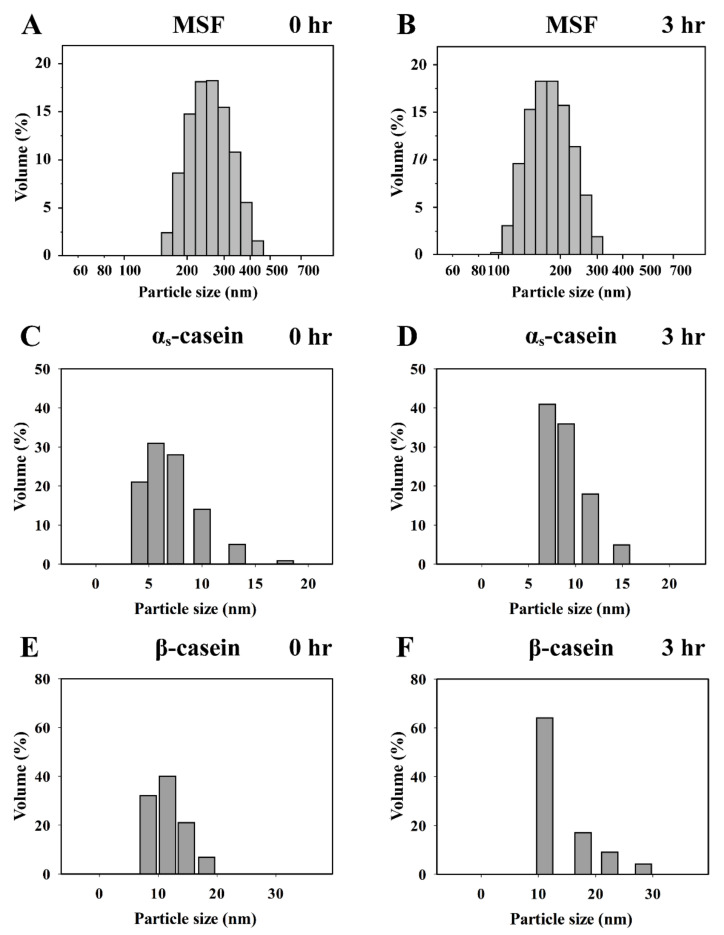
Particle size analysis of MSF, α_s_-CN and β-CN treated with chymosin (0.03 units/mL) at 30 °C for 0 and 3 h. (**A**) MSF for 0 h; (**B**) MSF for 3 h; (**C**) α_s_-CN for 0 h; (**D**) α_s_-CN for 3 h; (**E**) β-CN for 0 h; (**F**) β-CN for 3 h.

Skim milk and individual milk protein samples with 0.03 units of chymosin/mL were incubated at 30 °C for 0 and 3 h, respectively. The resulting MSF and individual milk protein samples were analyzed by a nanoparticle analyzer (Figure 5). The particle size of MSF with chymosin was 180–460 nm after 0 h incubation, while the mean particle size of MSF with chymosin was 254.4 nm after 0 h of incubation (Figure 5A). De Kruif and Holt (2003) reported that the normal particle size of casein micelles is approximately 50–600 nm, and the mean particle size is approximately 200 nm [30]. Huppertz et al. (2018) reported that casein micelles consisting of β-CN, κ-CN, and α_s_-CN are highly hydrated particles. κ-CN exists on the surface of casein micelles, and β-CN and α_s_-CN are predominantly found in the interior [4]. In solution, the milk protein spreads to form a polyelectrolyte layer, such as a hairy layer, which brings sterics and charges repulsion between protein particles, providing stability. When the casein micelles are linked with chymosin, the polyelectrolyte layer of the casein micelles is hydrolyzed while causing the casein micelles to become unstable and precipitate out of solution. The particle size of the MSF with chymosin was 92–316 nm after 3 h of incubation, while the mean particle size of the MSF with chymosin was 179.2 nm after a 3 h incubation period (Figure 5B). This is because only whey proteins such as β-LG and some casein micelles remained in the MSF, and it decreased the mean particle size of the MSF. The outcome revealed that β-CN, κ-CN, α_s_-CN, and some β-LG were trapped in the curds, and the MSF mostly consisted of whey protein and some casein micelles.

The effects of chymosin (0.03 units/mL) on the hydrolysis of individual β-CN and α_s_-CN samples at 30 °C for 0 and 3 h were also investigated, however, no significant changes were observed in either. The particle sizes of α_s_-CN with chymosin were 4.2–17.8 nm and 7.0–14.9 nm after 0 h and 3 h incubation, respectively (Figure 5C,D). The particle sizes of β-CN with chymosin were 8.8–18.2 nm and 11.1–28.4 nm after 0 h and 3 h incubation, respectively (Figure 5E,F). We observed that the molecular weights of β-CN and α_s_-CN decreased after chymosin hydrolysis; however, there was no difference in particle size distribution. These results suggest that chymosin induced the hydrolysis of β-CN and α_s_-CN, but it did not cause aggregation of β-CN and α_s_-CN.

### 3.5. Hydrolysis Reaction Scheme on Casein Micelles and Individual Caseins Induced by Chymosin

**Figure 6:**
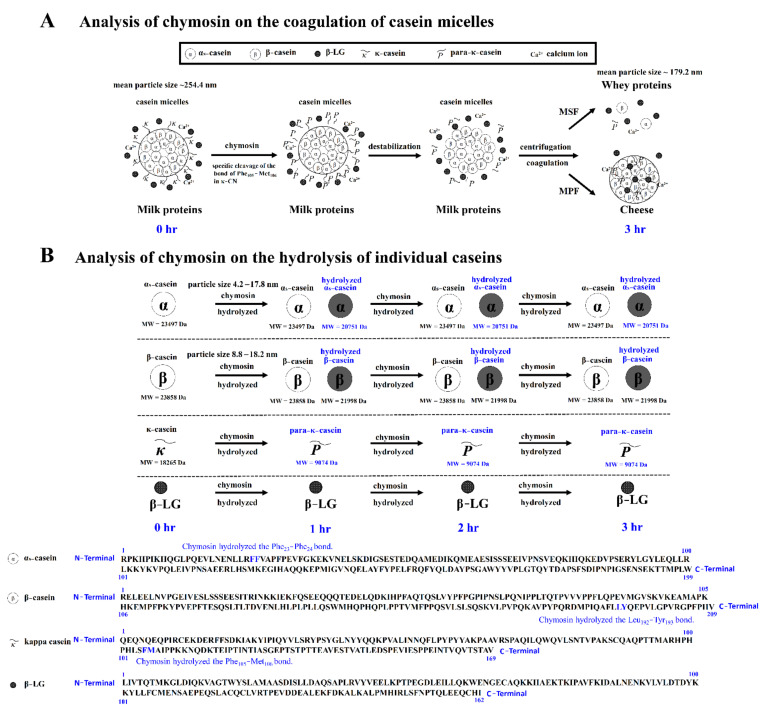
Hydrolysis reaction scheme on the casein micelles and individual caseins induced by chymosin. (**A**) casein micelles; (**B**) individual caseins.

Chymosin hydrolyzes the specific peptide bond of κ-CN, leading to destabilization and aggregation of casein micelles. It has a specific function during the bioactive reaction in cheese-making. According to our results, a hydrolysis reaction scheme on the casein micelles and individual caseins induced by chymosin in the cheese-making process is shown in Figure 6. As shown in Figure 6A, the coagulation reaction on casein micelles induced by chymosin consists of two steps. κ-CN was surrounded by the external area of casein micelles. First, chymosin hydrolyzes κ-CN to form caseinomacropeptide and para-κ-CN in the external area of casein micelles [18]. The Phe105-Met106 bond of κ-CN was hydrolyzed by chymosin, resulting in the instability of the casein micelles. Then, the aggregation of unstable casein micelles was induced by chymosin. Hydrophobic interactions are an important impetus for coagulation and are indirectly enhanced by calcium ions in milk. These unstable casein micelles and a part of whey protein, such as β-LG, are then thought to be trapped to form cheese curds after 3 h of incubation. During the coagulation process, the mean particle size of MSF with chymosin decreased from 254.4 nm to 179.2 nm. The hydrolysis reaction scheme on the individual caseins induced by chymosin in the cheese-making process is shown in Figure 6B. The peptide bonds in β-CN, κ-CN, and α_s_-CN were hydrolyzed by chymosin, and this protease hydrolyzed the Leu192-Tyr193, Phe105-Met106, and Phe23-Phe24 bonds in α_s_-CN and β-CN, respectively [26]. Chymosin hydrolyzed most κ-CN after 1 h of incubation, while some β-CN and α_s_-CN were hydrolyzed by chymosin after 1 h of incubation. However, β-LG was not hydrolyzed by chymosin after 3 h of incubation. These hydrolyzed β-CN, κ-CN, α_s_-CN, and β-LG were entrapped into the cheese curd.

## 4. Conclusions

We analyzed the effect of chymosin on the physicochemical and hydrolysis characteristics of casein micelles and individual caseins. SDS-PAGE analysis showed that milk proteins, including β-CN, κ-CN, α_s_-CN, and some β-LG, were coagulated by chymosin. In the catalysis process, chymosin hydrolyzed β-CN, κ-CN, and α_s_-CN but not β-LG. The hydrolysis of κ-CN by rennet occurred earlier than the hydrolysis of β-CN and α_s_-CN. MALDI-TOF-MS analysis indicated a decrease in the molecular weights of the hydrolyzed β-CN and α_s_-CN. Particle size analysis suggested that there was no difference in particle size distribution between β-CN and α_s_-CN after hydrolysis. Moreover, β-LG and hydrolyzed β-CN, κ-CN, and α_s_-CN were entrapped in the cheese curd.

## Data Availability

The data presented in this study are available on request from the corresponding author.
